# Tumor Suppressor RASSF1A Promoter: p53 Binding and Methylation

**DOI:** 10.1371/journal.pone.0017017

**Published:** 2011-02-25

**Authors:** Yihao Tian, Yu Hou, Xiang Zhou, Hanhua Cheng, Rongjia Zhou

**Affiliations:** Department of Genetics and Center for Developmental Biology, College of Life Science, Wuhan University, Wuhan, China; Instituto Nacional de Câncer, Brazil

## Abstract

Oncogenes and tumor suppressors work in concert to regulate cell growth or death, which is a pair of antagonist factors for regulation of tumorigenesis. Here we show promoter characteristic of tumor suppressor *RASSF1A*, which revealed a p53 binding site in the distal and a GC-rich region in the proximal promoter region of RASSF1A, in despite of TATA box-less. The GC-rich region, which is ∼300 bp upstream from the *RASSF1A* ATG, showed the strongest promoter activity in an assay of *RASSF1A*-driving *GFP* expression. Methylation analysis of the CpG island showed that 78.57% of the GC sties were methylated in testis tumor samples compared with methylation-less in normal testis. Hypermethylation of the GC-rich region is associated with RASSF1A silencing in human testis tumors. In addition, electrophoretic mobility shift assay indicated that p53 protein bound to the RASSF1A promoter. Further chromatin immunoprecipitation confirmed p53 binding to the RASSF1A. Moreover, p53 binding to the promoter down-regulated RASSF1A expression. These results suggest that p53 protein specifically binds to the RASSF1A promoter and inhibits its expression. Our results provide new insight into the mechanism of action of tumor suppressors and may be a starting point for development of new approaches to cancer treatment.

## Introduction

Cancer is a leading cause of death worldwide, accounting for 7.9 million deaths in 2007 (around 13% of all deaths) (WHO 2007, http://www.who.int/mediacentre/factsheets). Cancer is the number one killer of people in cities of Chin (25% of all deaths) based on recent census (MOH 2008, www.moh.gov.cn). Furthermore, deaths from cancer worldwide are projected to continue rising, with an estimated 12 million deaths in 2030. On the cellular level, oncogenes and tumor suppressors are key factors in controlling cell fate. Balance between them is essential for cell growth, differentiation and death.

Among tumor suppressors, p53 is the first and well-known tumor-suppressor gene, which plays a central role in eliciting cellular responses to a variety of stress signals, such as DNA damage, through regulation of cell-cycle arrest, senescence or apoptosis to prevent the development of cancer [1], [2]. Other cellular processes are also regulated by the p53 protein, including autophagy [3] and exosome secretion [4]. There is evidence that p53 protects the integrity of the genome by decreasing the levels of intracellular reactive oxygen species; this reduces oxidative damage and allows repair of genomic damage that might otherwise lead to the acquisition of oncogenic mutations [5], [6]. The action of p53 as a tumor suppressor is mainly due to its transcription factor activity, which can activate or repress downstream targets. Recently, a p53-cofactor JMY has been identified as a transcriptional co-activator of p53 [7]. To date, 129 p53-responsive genes have been identified that encode proteins, which mediate a wide variety of cellular stress responses [3]. The microRNAs miR-34a and miR-34b/c are also p53 target genes that may mediate the induction of apoptosis, cell cycle arrest and senescence [8]. In addition, posttranslational modifications of p53, including phosphorylation, methylation, acetylation, sumoylation, neddylation and glycosylation, may regulate p53 transcriptional activity and stability [9]. Recently, quaternary architecture of p53 was also revealed by structure biology studies [10]. Moreover, an increasing variety of proposed mechanisms through which p53 functions suggests further complexity of p53 [11], [12].

It is not well understood how p53 contributes to the activation of cell death and which factors determine whether p53 induction triggers apoptosis and/or cell cycle arrest. Elucidation of new p53 pathways, especially identification of the p53 negative regulator MDM2 in cancer cells, has been useful in the development of targeted therapies [13]. Further insights into the p53 pathway and p53-mediated transcriptional control may provide a better understanding of the molecular mechanisms underlying p53-mediated tumor suppression.

The tumor suppressor RASSF1A (Ras association domain family 1 isoform A) is an recently identified anti-cancer factor whose inactivation has been implicated in the development of many human cancers [14], [15], [16], [17]. Loss of RASSF1A expression stemming from RASSF1A point mutations and promoter hypermethylation is one of the most common events in many types of human cancers [18]. RASSF1A-knockout mice are prone to spontaneous and induced tumorigenesis [19], [20]. However, the mechanism of RASSF1A tumor suppression and RASSF1A pathways are not well understood, although K-RAS may regulate a pro-apoptotic pathway by binding to RASSF1A, which is a RAS effector [21], [22]. When bound to GTP, activated RAS regulates cellular responses through RAS effector proteins and their complex signal transduction cascades, such as those mediated by the Raf serine/threonine kinases, PI3-K and RASSF1A [21], [22]. Emerging evidence suggests that RASSF1A is a microtubule-binding protein that stabilizes microtubules [23], maintains genomic stability by modulating tubulin dynamics [24], [25], [26] and regulates cell cycle arrest and mitotic progression [27], [28], [29]. Thus, RASSF1A is potentially an important diagnostic and therapeutic target for the development of new anticancer drugs.

Here we present an unexpected finding of antagonism between two tumor suppressors p53 and RASSF1A, which may reflect inherent balance between cell death and survival this pair of tumor suppressors regulate in cancer cells.

## Materials and Methods

### Cells and tissues

COS-7 and the human cervical carcinoma cell lines Siha were obtained from China Centre for Type Culture Collection (Wuhan, China). Testis samples were provided by Ren-Min Hospital, Wuhan University.

### Plasmids and constructs

pRASSF1A-GFP vector. To generate EGFP-tagged *RASSF1A* promoter vectors (pRASSF1A-GFP), three portions (3173 bp, 1022 bp, 300 bp) of the *RASSF1A* promoter were amplified by PCR and subcloned into the *Hind*III and *Kpn*I sites of the pEGFP-1 mammalian expression vector respectively (BD Biosciences Clontech, Palo Alto, CA, USA). The primer sequences were as follows: sense primers 5′-GGAAAGCTTTGGGTCTGGAATAGTTTAGGG-3′ (3173 bp, (construction c), 5′-GCAAAGCTTGCGGCTGCAGGCGCGAAG-3′ (1022 bp, construction b) and 5′- GCAAAGCTTTATCTCCGCGTGGTGCTT-3′ (300 bp, construction a); and antisense primer 5′-CCCGGTACCGGCCCGGTTGGGCCCGTGCT-3′. PCR cycling conditions were as follows: 5 min at 94°C, 35 cycles of 40 s at 94°C, 40 s at 62°C and 3 min at 72°C. All constructs were confirmed by sequencing.

HIS–p53. The full-length p53 coding region (1179 bp) was PCR-amplified from the pRK-7-Flag-Neo-Insert vector (a generous gift from Dr. Shu hongbin (Wuhan University), which contains the full length of p53 coding sequence, using the p53-1 primer (5′-CAGGGATCCATGGAGGAGCCGCAGTCAG-3′) and the p53-2 primer (5′-AGAGAATTCGTCTGAGTCAGGCCCTTCT-3′). The PCR product was cloned in-frame into the prokaryotic expression vector PET32a (Amersham Biosciences, Uppsala, Sweden) using the BamHI and EcoRI (HIS–p53) sites.

### Sodium bisulphate modification and DNA methylation analysis

Frozen and paraffin-embedded testis tumor samples were digested by proteinase K (0.5 mg ml^−1^) in Tris (10 mM, pH 8.0), 1% SDS, 0.45% Tween 20 and EDTA (0.1 M, pH 8.0), followed by standard phenol-chloroform extraction. Genomic DNA was recovered after ethanol precipitation. Sodium bisulphate modification was performed as follows: 2 µg of genomic DNA was denatured by boiling for 10 min at 100°C and incubated in 0.3 M NaOH for 15 min at 37°C. Cytosine residues were sulphonated in 3.12 M sodium bisulphate (pH 5.0, Sigma, St Louis, MO, USA) and 5 mM hydroquinone (Sigma) in a thermocycler for 18 h at 55°C. Modified DNA was purified using the Wizard DNA clean-up system (Promega, Madison, WI, USA). The conversion reaction was completed by desulphonating in 0.3 M NaOH for 10 min at room temperature. The DNA was ethanol precipitated and resuspended in 50 µl of distilled water. Methylation-specific PCR (MSP) was then used to assess the methylation status of the promoter regions of the RASSF1A gene. Methylated specific primers: sense primer (5′-CGAGAGCGCGTTTAGTTTCGTT-3′) and antisense primer (5′-CGATTAAACCCGTACTTCGCTAA-3′) were added to reaction buffer (10 µl) containing 2 µl dNTPs (2 mM) and 1 U Taq polymerase at 94°C for 25 s, 58°C for 20 s and 72°C for 20 s for 40 cycles. We performed semi-nested PCR using the antisense primer 5′-GCTAACAAACGCGAACCG-3′. Unmethylation-specific primers were as follows: sense primer (5′-GGTTTTGTGAGAGTGTGTTTA-3′) and antisense primer (5′-CACTAACAAACACAAACCAAAC-3′). The PCR products were purified using a gel extraction kit (Promega), ligated into the T-easy vector (Promega) and sequenced.

### Cell culture and transfection

COS-7 and Siha cells were grown in RPMI1640 medium containing 20% fetal bovine serum (HyClone, Logan, Utah, USA) at 37°C with 5% CO_2_. Medium was replaced every other day. For transient transfection, cells were seeded onto 24-well plates at a density of 10^5^cells/well. Constructs were transfected into COS-7 or Siha cells with Lipofectamine™ 2000 (Invitrogen, Carlsbad, CA, USA) according to the manufacturer's instructions. After 48 h of incubation, cells were collected and used for analysis.

### Immunohistochemistry

Serial 7-µm sections were cut with a cryostat (Leica, Bensheim, Germany). Briefly, the sections were deparaffined in xylene and alcohol, fixed in methanol at −20°C for 20 min and endogenous peroxide activity in the samples was quenched using 3% hydrogen peroxide for 20 min at room temperature. After rinsing with PBS, nonspecific antibody binding was blocked by incubating the sections for 20 min in goat serum. The samples were then incubated with antibodies in a moist chamber overnight at 4°C. The expression of RASSF1A proteins was analyzed using anti-RASSF1A (eBioscience, CA, USA), using the SABC and DAB visualization methods according to the manufacturer's instructions (Boster Company, China).

### Western blots

For western blots, Siha cells were lysed in ice-cold buffer and incubated on ice for 10 min. The samples were boiled in loading buffer for 10 min and centrifuged at 13000×g for 20 min at 4°C, and supernatants were collected. Equal amounts of protein were loaded into 12% polyacrylamide gels, electrophoresed and the separated proteins were transferred to polyvinylidene difluoride (PVDF) membranes. Nonspecific antibody binding was blocked with 5% BSA in Tris-buffered saline (TBS) for 1 h at room temperature. The membrane was then incubated with human anti-p53 (1∶500, Santa Cruz Biotech, CA, USA), anti-RASSF1A (1∶500), or anti-β-actin (1∶1000, Santa Cruz Biotech, CA, USA) antibody overnight at 4°C, followed by HRP-conjugated secondary antibody (1∶50000, Santa Cruz Biotech, CA, USA) for 1 h. The immunoreactive signal was revealed by ECL reagent (Pierce, Rockford, USA).

### Electrophoretic mobility shift assays (EMSA)

Recombinant HIS–p53 fusion protein was purified from cell lysates by imidazole using HiTrap affinity columns according to the manufacturer's instruction (Amersham Biosciences). In order to testify that p53 is a potential transcription factor of RASSF1A promoter, DNA binding assay was employed. For the EMSA assay, two probes were used: a wild type probe and a mutant probe. The wild type probe sequence is 5′-TTCCGGGTCAGGGCCTGGCAGGAAGGGAGAT-3′, and that of the mutant probe is 5′-TTCCGGGTTGAAGCCTGGCAGGAAGGGAGAT-3′. Double-stranded probes were made by annealing oligonucleotides into double strands and labelled with T4 polynucleotide kinase (Gel Shift Assay Core System; Promega) in the presence of [γ-32P]dATP. Unincorporated nucleotides were removed by chromatography through Sephadex G-25 resin. The concentrations for the DNA binding reaction were 1.5 µM for protein and 40 nM for DNA. The binding reaction was carried out at room temperature for 1 h according to the manufacturer's protocol (Gel Shift Assay Core System; Promega) before loading onto a 6% polyacrylamide gel. Complexes were separated by native gel electrophoresis (10 V/cm at 4°C with 0.5×TBE buffer). The antibody or competitor oligonucleotide (the single stranded oligonucleotide in 20-fold molar excess) was added at the start of the incubation for the supershift and competition experiments.

### Chromatin immunoprecipitation (ChIP)

Siha cells were cross-linked in 1% formaldehyde to cross-link endogenous proteins and DNA. Samples of sonicated chromatin were immunoprecipitated with no antibody (beads only), preimmuno IgG (IgG), anti-p53 antibody, and Protein G PLUS-Agarose (Santa Cruz Biotech, CA, USA) was added to the samples. DNA isolated from the immunoprecipitated complex was amplified by PCR with primers flanking the p53 binding site (a region of 147 bp corresponding to the −2799 to −2653of the RASSF1A promoter). The primer sequences are: sense primer 5′-GCCTCCATCATCAACCTCT-3′ and the antisense primer 5′-GAGGGAAACTTTCTGTGTC-3′. The amplified PCR fragments were analysed on 2% agarose gel. A region of 192 bp flanking intron 1 and exon 2 of the RASSF1A was amplified as a control with primers: sense: 5′-TGAGGTAACCCACTGAGATAGG-3′ and antisense: 5′-CGCAACAGTCCAGGCAGA-3′.

## Results

### Hypermethylation of the GC-rich region of the *RASSF1A* promoter is associated with *RASSF1A* silencing in human testis tumors

**Figure 1:**
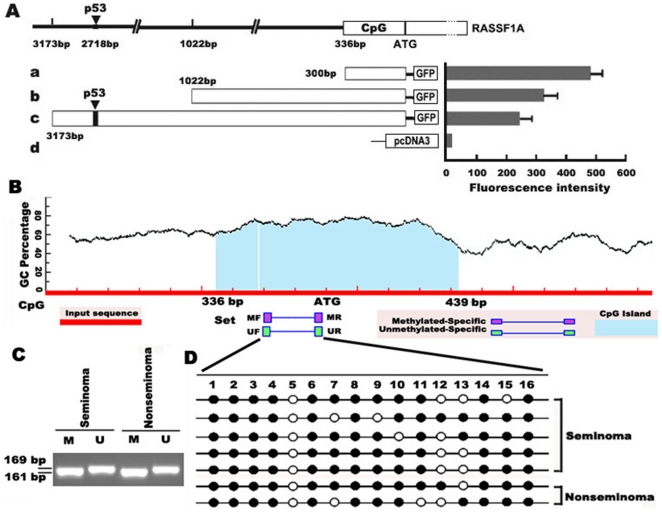
Promoter activity and methylation analysis of *RASSF1A* in human testis cancer. **A**, *RASSF1A* promoter activity by GFP expression under the control of different length of RASSF1A promoter segments. A predicted p53 binding site in *RASSF1A* promoter (−2718 bp) was showed in the construction c. **B**, CpG island prediction of *RASSF1A* by software MethPrimer online (http://www.urogene.org/methprimer/). Predicted CpG island is indicated by blue color. **C**, Representative results of the methylation-sensitive PCR (MSP) analysis of *RASSF1A* in both seminoma and nonseminoma. M, MSP PCR; U, unmethylation-sensitive PCR. **D**, DNA methylation status of individual CpG sites by sodium bisulfite sequencing analysis. Black and white circles represent methylated and unmethylated CpGs respectively.

**Figure 2:**
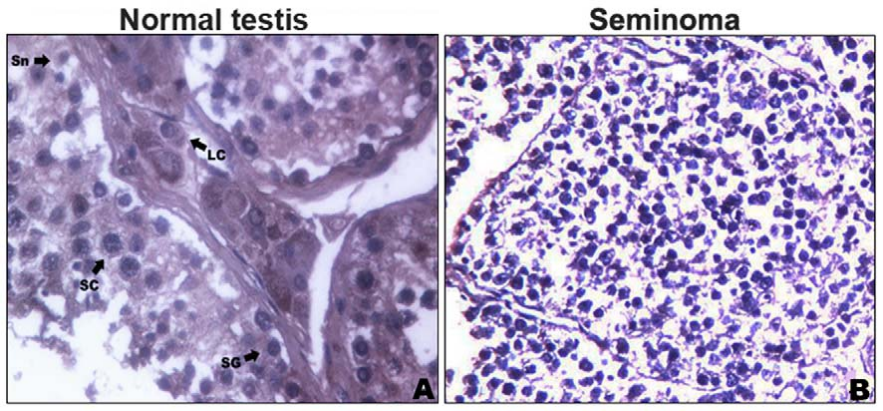
Immunohistochemical staining of RASSF1A in human testis cancer. Normal testis sample (**A**) and testis tumor with methylated RASSF1A (**B**). Normal testis section stained positively for RASSF1A. RASSF1A was mainly expressed in cytoplasm of the spermatogonia (SG), spermatocytes (SC), Sertoli cells (Sn) and Leydig cells (LC) of normal testis, while seminoma sample with methylated RASSF1A showed weak staining. Nuclei were stained with Hematoxlin.

To get insight into regulation of *RASSF1A* expression, we first analyzed its promoter characteristic, which revealed a p53 binding site and a GC-rich region. The GC-rich region, which is ∼300 bp upstream from the *RASSF1A* ATG, showed the strongest promoter activity in an assay that tested the ability of promoter regions to drive GFP expression (Fig. 1A). *RASSF1A* lacks a TATA box in its promoter, and the GC-rich region seems essential for transcription initiation by the RNA polymerase II complex; this is consistent with recent findings that transcription over short distances (250 nucleotides upstream) is common for active promoters [30]. This region is also within the CpG island of the *RASSF1A* promoter (Fig. 1B). Further bisulphite sequencing of the *RASSF1A* CpG island showed that 78.57% of the GC sties (11/14) were methylated in testis tumors in both seminoma and nonseminoma samples while no methylation was detected in normal testis (Fig. 1C, D). Immunohistochemical analysis revealed that high methylation resulted in very weak expression of RASSF1A in testis tumor samples (Fig. 2B); while RASSF1A was expressed in both the nuclei and cytoplasm of spermatogonia, spermatocytes, Sertoli cells and Leydig cells in normal testis (Fig. 2A).

### p53 binds to *RASSF1A* promoter

**Figure 3:**
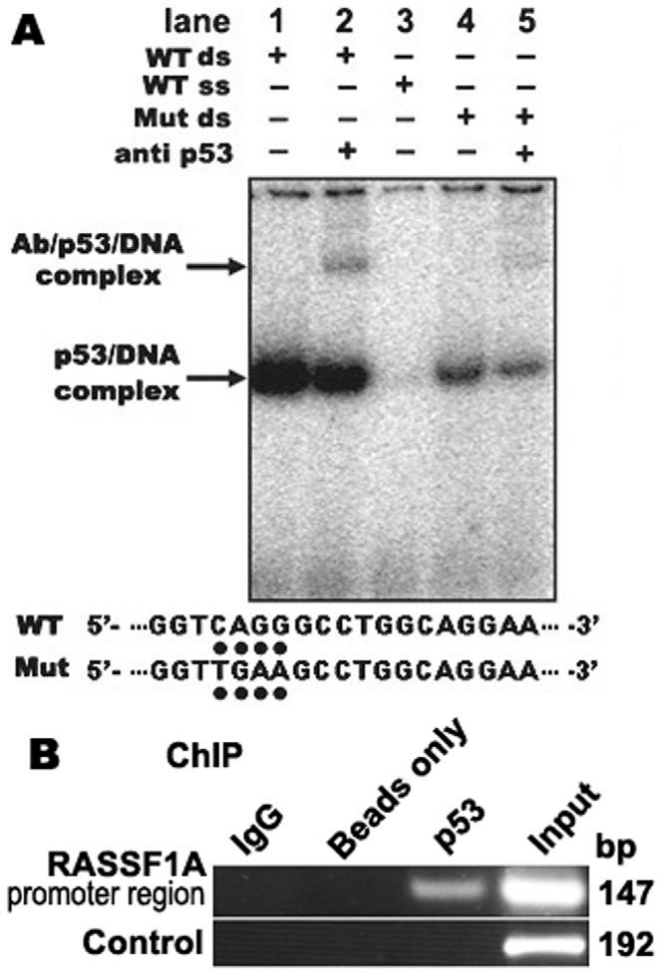
p53 protein binds to the *RASSF1A* promoter. **A**, EMSA analysis of p53 binding to the *RASSF1A* promoter. WT, the wild type p53 binding site probe from human *RASSF1A* promoter; Mut, mutant p53 binding site probe; ds, two complementary oligonucleotide probes; ss, single stranded probe. The supershift band in lane 2 contains the anti-p53/wt DNA/p53 protein complex. Complexes were separated by native gel electrophoresis. Mutant sequence was showed in lower panel. **B**, ChIP assay of p53 binding to the *RASSF1A* promoter in Siha cells. Samples of sonicated chromatin from Siha cells cross-linked in 1% formaldehyde were immunoprecipitated with anti-p53 antibody, preimmuno IgG (IgG) and no antibody (beads only). DNA isolated from immunoprecipitated material was amplified by PCR with primers to amplify the 147 bp *RASSF1A* promoter sequences flanking the p53 binding site (−2799 bp to −2653 bp). A region of 192 bp flanking intron 1 and exon 2 of the *RASSF1A* was amplified as a control. Input lanes represent sonicated chromatin samples as positive control. The amplified PCR fragments were analysed on 2% agarose gel.

As a predicted p53-binding site exists in the RASSF1A promoter (at −2718 bp), although lack of methylation CpG site in the binding site, further functional test for the binding site using gel-shift assay showed that His–p53 specifically and efficiently bound to *RASSF1A* (Fig. 3A). The specific interaction was confirmed by adding the p53 antibody into the binding reaction, resulting in a supershift band (antibody/p53/RASSF1A) (Fig. 3A, lane 2). Furthermore, a mutant RASSF1A probe decreased the formation of the p53/RASSF1A complex. Further test *in vivo* of p53 binding to the RASSF1A promoter using Chromatin Immunoprecipitation (ChIP) assay confirmed p53 binding to the RASSF1A (Fig. 3B). These results indicated that the p53 protein specifically binds to the RASSF1A promoter.

### p53 binding to *RASSF1A* promoter downregulates *RASSF1A* expression

**Figure 4:**
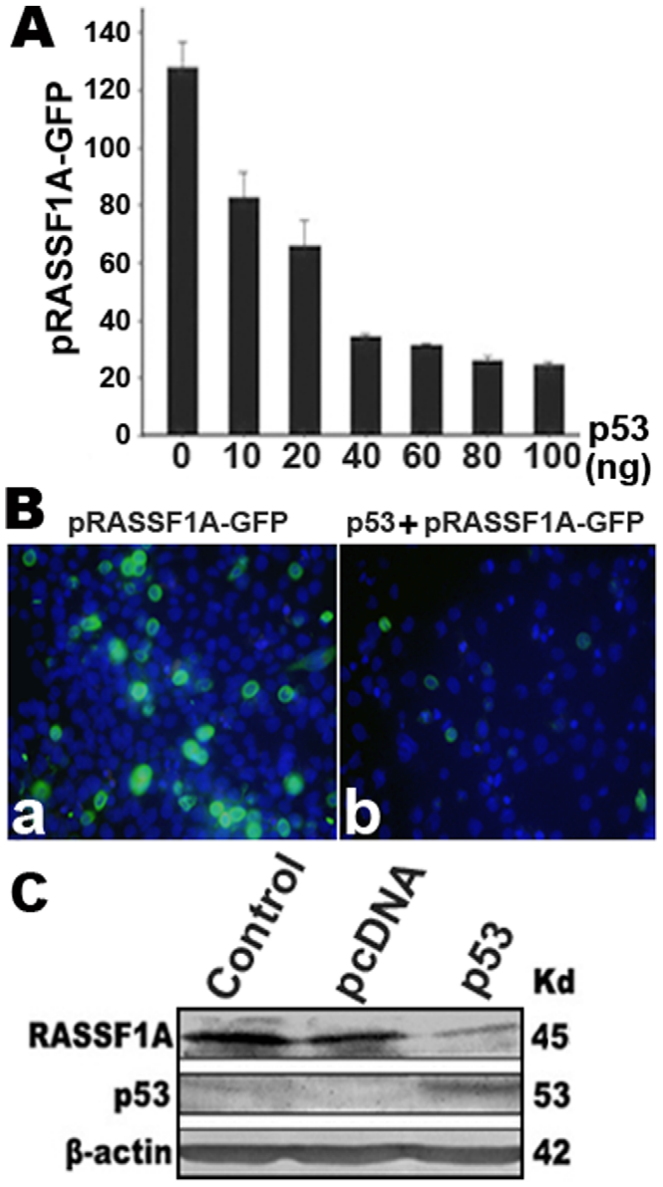
Down-regulation of *RASSF1A* by p53. **A**, *RASSF1A* promoter activity was inhibited by p53 protein with a dose dependent manner. pRASSF1A-GFP (construction c in Fig. 1) was co-transfected with different concentration of CMV-p53 into COS-7 cells, and transfected cells were analyzed by the flow cytometry. **B**, pRASSF1A-GFP was transfected into COS-7 cells (**a**) or co-transfected with p53 (**b**), and images were taken under fluorescent microscopy. p53 remarkably decreased expression of RASSF1A-GFP. GFP was mainly expressed in the cytoplasm. Nuclei were stained with Hoechst. **C**, Overexpression of p53 in Siha cells decreased RASSF1A protein levels, revealed by Western blot analysis of RASSF1A and p53 expression using anti-RASSF1A or p53 antibody after transfection of the p53 expression plasmid into Siha cells. Lysates of non-transfected cells and cells transfected with the pcDNA3 vector were used as controls. β-actin was used as an internal control. Molecular weights of the proteins are shown on the right.

We then examined the effect of p53 binding on RASSF1A expression using a RASSF1A promoter-EGFP expression reporter construct (pRASSF1A-EGFP). When p53 and pRASSF1A-EGFP were co-transfected into COS-7 cells, expression of the reporter EGFP was significantly inhibited in a dose-dependent manner, reaching saturation when 40 ng of the p53 vector was transfected (Fig. 4A). Fluorescent microscopy analysis showed directly that p53 remarkably decreased expression of RASSF1A-GFP (Fig. 4B). Further, when p53 was overexpressed in endogenous RASSF1A-expressing Siha cells, RASSF1A protein expression was markedly decreased (Fig. 4C). These results indicated that p53 protein not only specifically binds the RASSF1A promoter but also efficiently downregulates RASSF1A expression.

## Discussion

Tumor suppressors restrict the proliferation of abnormal or cancerous cells by regulating cell-cycle arrest, senescence or apoptosis to prevent the development of cancer. Loss of a single tumor suppressor is sufficient for initiation of tumorigenesis. Tumor suppressors can also act together using a common pathway to regulate cell growth [31]. However, it remains unclear whether or how tumor suppressors regulate each other against tumorigenesis.

In this study, we showed that the p53 tumor suppressor inhibits RASSF1A by binding to its promoter. The crosstalk of tumor suppressors p53 and RASSF1A reveals a new pathway for cancerous cells to regulate cell death. This could be an inherent feedback loop for cell survival when stress or damage is present. When p53 was upregulated, we observed marked inhibition of RASSF1A expression, due to p53 binding to the RASSF1A promoter. Interestingly, recent reports showed that another member of the RASSF family, RASSF5, regulates growth inhibition via promoting p53 nuclear localization [32], which may provide a clew for a common link between RASSF and p53 for cell death regulation.

Physiologically, the apoptotic response reflects the cumulative or integrative action of numerous p53-induced signals. On the one hand, p53 regulates the cell cycle and apoptosis by pathways such as MDM2-p53 and microRNAs-p53, or by inducing the expression of numerous apoptotic genes that contribute to the activation of the death receptor and mitochondrial apoptotic pathways and by affecting the efficiency of survival signalling. On the other hand, and of particular note, the p53-RASSF1A pathway described here may attenuate cell damage by silencing the RASSF1A tumor suppressor. This reflects the inherent balance between the death of cancerous cells and cell survival, a balance regulated by this pair of tumor suppressors. The ability to engage apoptotic pathways via several routes is likely to be important for the activity of the tumor-suppressor p53. These findings add a new layer of complexity to what is known about the p53 network. In addition, hypermethylation of the proximal promoter region of *RASSF1A*, which we detected in testis tumors, is another common event for cancer cells to escape from apoptosis. Thus we conclude that the p53-RASSF1A pathway, at least along with hypermethylation (in the proximal promoter region of *RASSF1*) and point mutations, contribute to RASSF1A silencing in cancerous cells.

These results add to the known complexity of the p53 network, but also provide new insights into the p53-RASSF1A pathway and highlight the potential importance of crosstalk among tumor suppressors in the prophylaxis and treatment of cancer inherent antagonism. This may also provide a starting point for new strategies for improved cancer therapy using tumor suppressors as targets. Further analysis of the p53-RASSF1A pathway should provide a better understanding of the molecular mechanisms of tumor suppression.
